# Cognition-based variable admittance control for active compliance in flexible manipulation of heavy objects with a power-assist robotic system

**DOI:** 10.1186/s40638-018-0090-x

**Published:** 2018-11-12

**Authors:** S. M. Mizanoor Rahman, Ryojun Ikeura

**Affiliations:** 1 Department of Mechanical and Aerospace Engineering, Tandon School of Engineering, New York University (NYU), 6 MetroTech Center, Brooklyn, NY 11201 USA; 2 Department of Mechanical Engineering, Graduate School of Engineering, Mie University, Tsu, Mie 514-8507 Japan

**Keywords:** Human–robot collaboration, Collaborative flexible automation, Industrial robotics, Power-assist robot, Industrial object manipulation, Cognition, Variable admittance control, Active compliance, User-friendliness, Risk assessment, Safety

## Abstract

In the first step, a 1-DOF power-assist robotic system (PARS) is developed for lifting lightweight objects. Dynamics for human–robot co-manipulation of objects is derived that considers human cognition (weight perception). Then, admittance control with position feedback and velocity controller is derived using weight perception-based dynamics. Human subjects lift an object with the PARS, and HRI (human–robot interaction) and system characteristics are analyzed. A comprehensive scheme is developed to evaluate the HRI and performance. HRI is expressed in terms of physical HRI (maneuverability, motion, safety, stability, naturalness) and cognitive HRI (workload, trust), and performance is expressed in terms of manipulation efficiency and precision. To follow the guidance of ISO/TS 15066, hazard analysis and risk assessment are conducted. A constrained optimization algorithm is proposed to determine the values of the control parameters that produce optimum HRI and performance with lowest risk. Results show that consideration of weight perception in dynamics and control helps achieve optimum HRI and performance for a set of hard constraints. In the second step, a weight perception-based novel variable admittance control scheme is proposed as an active compliance to the system, which enhances the physical HRI, trust, precision and efficiency by 53.05%, 46.78%, 3.84% and 4.98%, respectively, and reduces workload by 35.38% and thus helps achieve optimum HRI and performance for a set of soft constraints. The risk reduces due to the active compliance. Then, effectiveness of the optimization and control algorithms is validated using a multi-DOF PARS for manipulating heavy objects, and intuitive and natural HRI and performance for power-assisted heavy object manipulation are achieved through calibrating HRI and performance with that for manipulation of lightweight object.

## Introduction

Human workers need to handle heavy objects and materials (e.g., assembly parts, cartons, bulk materials in bags) in various industries such as construction, manufacturing and assembly, mining, transport and logistics, rescue and disaster operations, military operations and timber/forestry. However, manual manipulation is tedious and it reduces efficiency and causes musculoskeletal disorders, back pains and injuries in workers [1]. On the contrary, autonomous manipulation is usually inflexible (or less flexible) and less adaptable [2]. By flexible manipulation, we here mean the manipulation method that can be easily modified and reprogrammed to respond to altered circumstances or conditions, which is adaptable, adjustable and versatile [3]. By flexible manipulation, we do not mean manipulating an object that is itself flexible or deformable [4]. To achieve flexible manipulation, we propose that human-in-the-loop collaborative automation system such as the power-assist robotic system (PARS) can be comfortably used for object manipulation, where the combination of mechanical strength of a robot and intelligence of a human can make the human–robot system superior to a robot or an individual human [5]. PARSs can provide various advantages, e.g., (1) power assistance through sharing power and reducing haptically perceived heaviness, (2) flexibility in positioning and ease in motion control through direct human–machine haptic interface and haptic information sharing, (3) naturalness and intuitiveness as human intent is reflected through human input to the system, etc. These advantages can foster high precision, efficiency, robustness and human-friendliness in object manipulation [5, 6].

Reviews on state-of-the-art PARSs for industrial object manipulation show the early-stage works of Kazerooni that introduced the fundamental principles of information and power sharing of a PARS for load manipulation [5]. After that, a significant number of PARSs have been proposed for handling objects, e.g., [6–17]. In [6], Niinuma et al. proposed a power-assisted overhead crane for object manipulation and also compared its performance to that of conventional automated manipulation. In [7], Doi et al. proposed a pneumatically actuated hand crane-type PARS for object manipulation. Hara introduced a switching mechanism between automatic transfer and power-assist controls for horizontal manipulation of object [8]. Yagi et al. proposed a control method for a pneumatically actuated upper arm PARS for agricultural load manipulation [9]. Dimeas et al. introduced admittance neuro-control of a PARS for lifting objects [10]. Hara and Sankai demonstrated a “Hybrid Assistive Limb-HAL” prototype to assist humans in carrying heavy loads [11]. In [12, 13], it was shown how admittance controls were varied to adjust situations while handling large loads with power-assist. Various types of industrial assistive devices (IADs) for load manipulation were introduced and analyzed in [14]. In [15], Olivier et al. presented “Cobomanip”—an IAD for load manipulation in industries, and so forth. A few PARSs for object manipulation are already in practical applications in industries such as the “Power Loader Light-PLL” [16] and Cobot [17]. The above reviews show that power assisting automation technologies for manipulation of heavy objects achieved significant advancements. However, PARSs for object handling still have a few fundamental limitations or challenges as follows that require close attention:

### Mismatch between visual and haptic perceptions

A human user perceives reduced heaviness while manipulating an object with power-assist [5]. The user feed-forwardly estimates the manipulative force (load force, grip force) to manipulate (e.g., lift) the object with the PARS depending on visually perceived weight of the object [18]. Here, the load force reflects human’s intent in manipulation that influences the motion [5]. The haptically perceived weight is smaller than the visually perceived weight [5], and thus, the applied load force estimated by the user based on the visually perceived weight is incorrect (larger than the load force actually required to lift the object to the desired position successfully) that results in harmful motion (acceleration), poor safety and lack of stability [5, 19]. As a consequence, human–robot interaction (HRI) and overall performance in manipulation become unsatisfactory that also reduce user’s trust in the robot [20, 21]. Furthermore, cognitive workload and fatigue may increase if the user undergoes a careful visual check of the prospective weights before handling objects with power-assist to realize the difference between visually and haptically perceived weights.

Gravity compensation in robot dynamics can be an approach to solve the aforementioned problem [6, 10, 14]. However, zero gravity removes haptic feelings and restricts naturalness in direct kinesthetic co-manipulation of objects [22]. As an alternative approach, the gravity can be partly compensated by using a virtual mass in the dynamics [8, 12, 13]. In this approach, the mass value needs to be estimated in such a way that it provides expected haptic feelings in the user. However, basis of estimation of mass value for partial compensation of gravity has not been justified yet, which does not help achieve expected HRI and performance [19, 22]. Another alternative approach may be the use of a tentative feed-forward model of the load force as a user input to the PARS with a notion that the model may be adjusted if the user gains experiences [7, 8]. However, effectiveness of such notion has not been justified properly. Model-based predictive controllers (e.g., a model predictive controller—MPC) may also be used to generate the predicted input force based on an optimization scheme to provide predicted output (acceleration) [23]. Constant torque/force method [24] may also be used to provide constant or nearly constant output force/torque. However, the load force depends on object gravity in power-assist dynamics and the optimum input force provided by MPC or the constant force may not produce optimum haptic feelings in user. In fact, estimation of load force for manipulation with power-assist is a cognitive phenomenon that depends on user’s visual perception of object weight [5, 19], and hence, the input load force cannot be estimated by any computational model perfectly. Instead, the effects of excess in load force can be counterbalanced if an active compliance control method is proposed reflecting/mimicking user’s cognition in power-assist dynamics [19, 25]. Here, by cognition we mean human operator’s mental action or process of acquiring knowledge and understanding about the objects and environment through thoughts, experiences and senses [26]. Cognition can convey the similar meanings as perception, discernment, apprehension, learning, understanding, comprehension, insight, etc. Cognition can mean weight perception, which is the perception, recognition and discrimination of the heaviness of a lifted object [19]. It may be a combination of visual perception and haptic perception [27]. However, such cognitive or weight perceptual approaches integrating human thoughts, perception and capabilities are not observed with the state-of-the-art control strategies of PARSs except a few preliminary initiatives [19, 25, 28].

### Selection of appropriate control strategies

Selection of control strategies for manipulating objects with power-assist is very challenging [29]. Large inertia, friction and dynamic effects are expected while manipulating heavy objects, which can be compensated and positional accuracy can be provided by admittance controls [12]. Admittance parameters (e.g., virtual mass, damping and stiffness) can affect HRI and manipulation performance. For example, for large admittance parameters, large load force is required to move the object and the user feels more heaviness that may cause fatigue. The movement may also be slow due to low acceleration. However, it may be possible to achieve precise (e.g., smooth, fine) manipulation. On the contrary, low admittance parameters may need less human force to accelerate the object that may result in low fatigue, but precision in manipulation may reduce due to the reason that the robot is more reactive. These are the disadvantages of fixed admittance control that indicate the necessity of variable admittance control [13]. In [13], a variable admittance control strategy was proposed where a virtual mass varied to adjust acceleration and precision in power-assisted manipulation. However, the effects of excessive acceleration generating from user’s error in the programming of load force due to difference in perception between visual and haptic weights were not mitigated. Furthermore, changes in virtual mass (the mass value used in the dynamics) change acceleration [19], but it may also alter haptic perceptions [5]. Consequently, HRI and manipulation performance may be affected adversely [19]. Hence, a novel variable admittance control strategy seems to be necessary to modulate the kinematics (acceleration) and haptic perceptions differently in power-assisted manipulation to achieve better HRI and performance. However, such novel strategy has not been proposed and validated yet properly [19].

### Comprehensive evaluation scheme

A comprehensive evaluation scheme is necessary for PARSs for object manipulation, which can be used to optimize HRI and co-manipulation performance. Not only robotics parameters, but also HRI and manipulation performance need to be optimized to achieve human-friendliness in collaborative manipulation. Objective evaluation is emphasized; however, there are some HRI and performance criteria that can neither be measured objectively nor be ignored. Hence, subjective evaluation also needs to be considered as complementary to objective evaluation. HRI criteria should address both physical HRI (pHRI) and cognitive HRI (cHRI), and performance criteria should include the key performance indicators (KPIs) of power-assisted manipulation in actual industrial applications. The state-of-the-art literature shows a few detached initiatives for evaluation of PARSs. For example, only precision, stability and efficiency were evaluated in [6], and user comfort was evaluated in [9]. Safety in PARS was provided through mechanical design [12, 13, 30], but a safety evaluation and analysis method was not proposed. In [31], Sylla et al. focused on ergonomic criteria ignoring other HRI and performance criteria. Maurice et al. [32] addressed simulation-based musculoskeletal risks in power-assisted manipulation only. A complete hazard analysis and risk assessment for power-assisted manipulation is especially important due to the potential risk of unexpected motion [19]. Different standards and guidance such as ISO/TS 15066, ISO 10218–1 and ISO 10218-2 have been proposed to ensure safety of collaborative robotics [33–37]. However, initiatives to conduct risk analysis and ensure safety following this guidance for power-assisted manipulation have not been taken yet. As a result, it is still uncertain about simultaneous attainment of user-friendliness and high performance in power-assisted manipulation.

### Naturalness and intuitiveness in manipulation

The principle advantage of a PARS is that a user manipulates a heavy object with the PARS, but he/she feels lightweight [5]. For heavy and large objects manipulated with a PARS, the user cannot grasp the entire object using a power grip properly, and thus, the HRI and performance experienced for power-assisted manipulation of heavy objects may not reflect user’s naturalness and intuitiveness. However, appropriate methodology to achieve naturalness and intuitiveness in HRI and performance for power-assisted manipulation has not been proposed and validated yet.

**Fig. 1:**
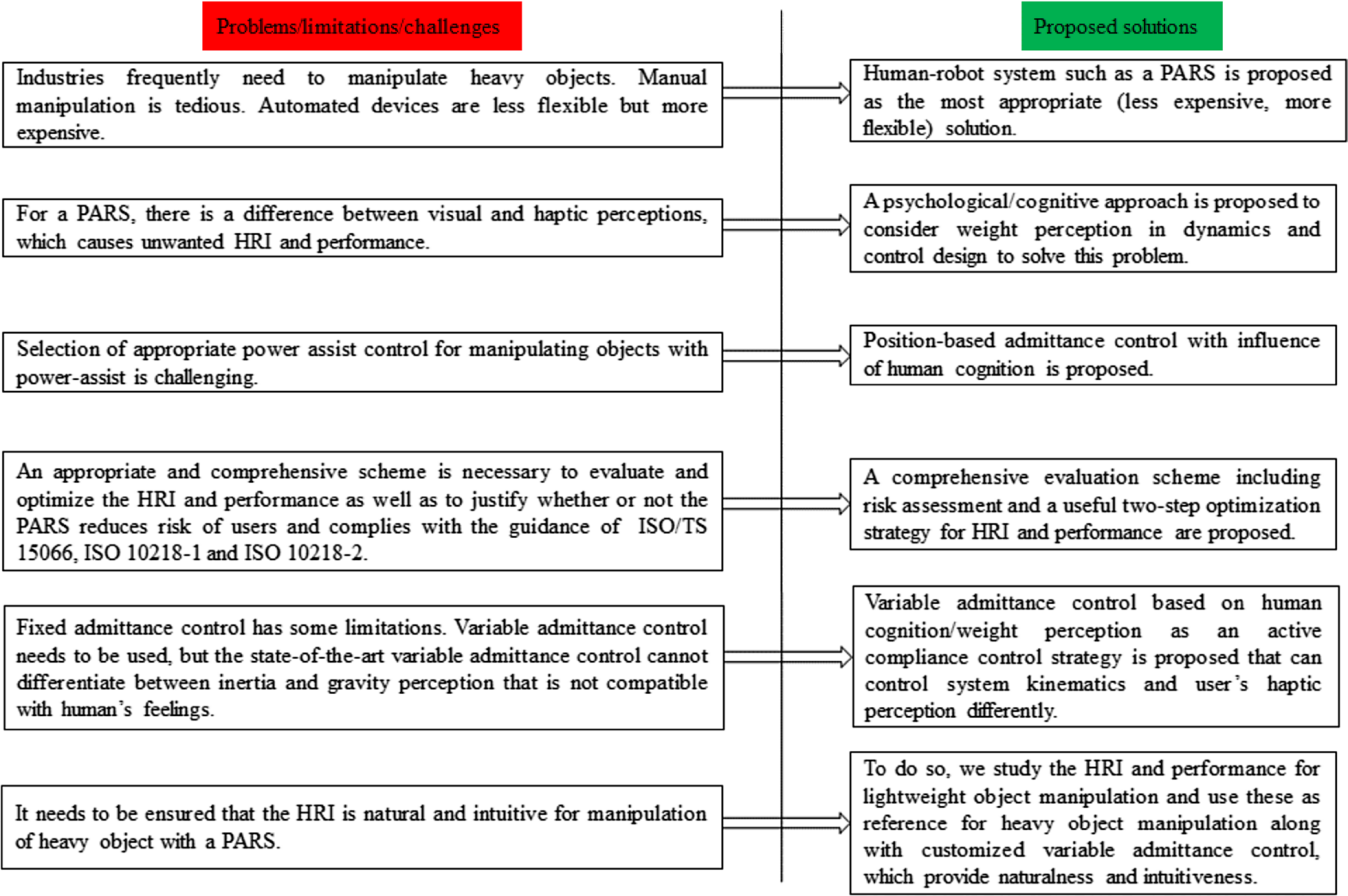
State-of-the-art problems/limitations/challenges of the PARSs for heavy object manipulation and the prospective solutions

Being motivated by the above limitations/challenges of the state-of-the-art PARSs for object manipulation, we summarized the specific problems of the state-of-the-art PARSs and proposed appropriate solutions as given in Fig. 1. According to the proposed solutions in Fig. 1, the purpose of this article is to develop a human-friendly PARS for industrial heavy object manipulation exploiting human cognition-based variable admittance control as a means of active compliance, develop a comprehensive scheme to evaluate the system, and ensure naturalness and intuitiveness in power-assisted manipulation. The core innovations as we attempt to bring are: (1) proposing appropriate control strategies for PARSs for object manipulation, (2) illustrating a method to include user’s weight perception in power-assist system dynamics and control, (3) proposing a comprehensive evaluation scheme including an HRI optimization method for power-assisted manipulation, and (4) proposing a method to achieve naturalness and intuitiveness in power-assisted manipulation.

To bring the core innovations as above, we adopt two main objectives for this article and use two steps to address the objectives:

i. In the first step (second section to seventh section), we investigate a method to include weight perception in the dynamics and derive weight perception-based fixed admittance control for the PARS. We then determine a comprehensive evaluation scheme including risk assessment and determine optimum HRI and performance for fixed admittance control using a local optimization scheme for a set of hard constraints for lifting lightweight objects with power-assist.
ii. In the second step (eight section to tenth section), we investigate a variable admittance control strategy based on weight perception, kinematics and kinetics features to add variable compliance to the PARS for improving HRI and performance so that optimum HRI and performance can be achieved for a set of soft constraints, and also risk in object manipulation is reduced. We evaluate the variable admittance control strategy for lifting lightweight objects. We then validate the optimization and control approaches for vertical lifting of heavy objects using a multi-DOF PARS. We calibrate the HRI and performance for heavy object manipulation through comparing the evaluation results for lightweight object manipulation with that for heavy object manipulation.

We then discuss how the findings can be used to develop power-assist devices to manipulate heavy materials in actual industrial environments. Note that as a preliminary initiative, we here consider vertical lifting only as it is common in industries, humans feel heaviness more in vertical lifting, and it needs more power assistance. We also explain how the proposed approach can be augmented to 6-DOF dexterous manipulation. The presented 1-DOF design seems to be simple, but we think that such design may be sufficient to address the objectives, and achieve the core innovations.

## Design and development of a simple PARS for lifting lightweight objects

**Fig. 2:**
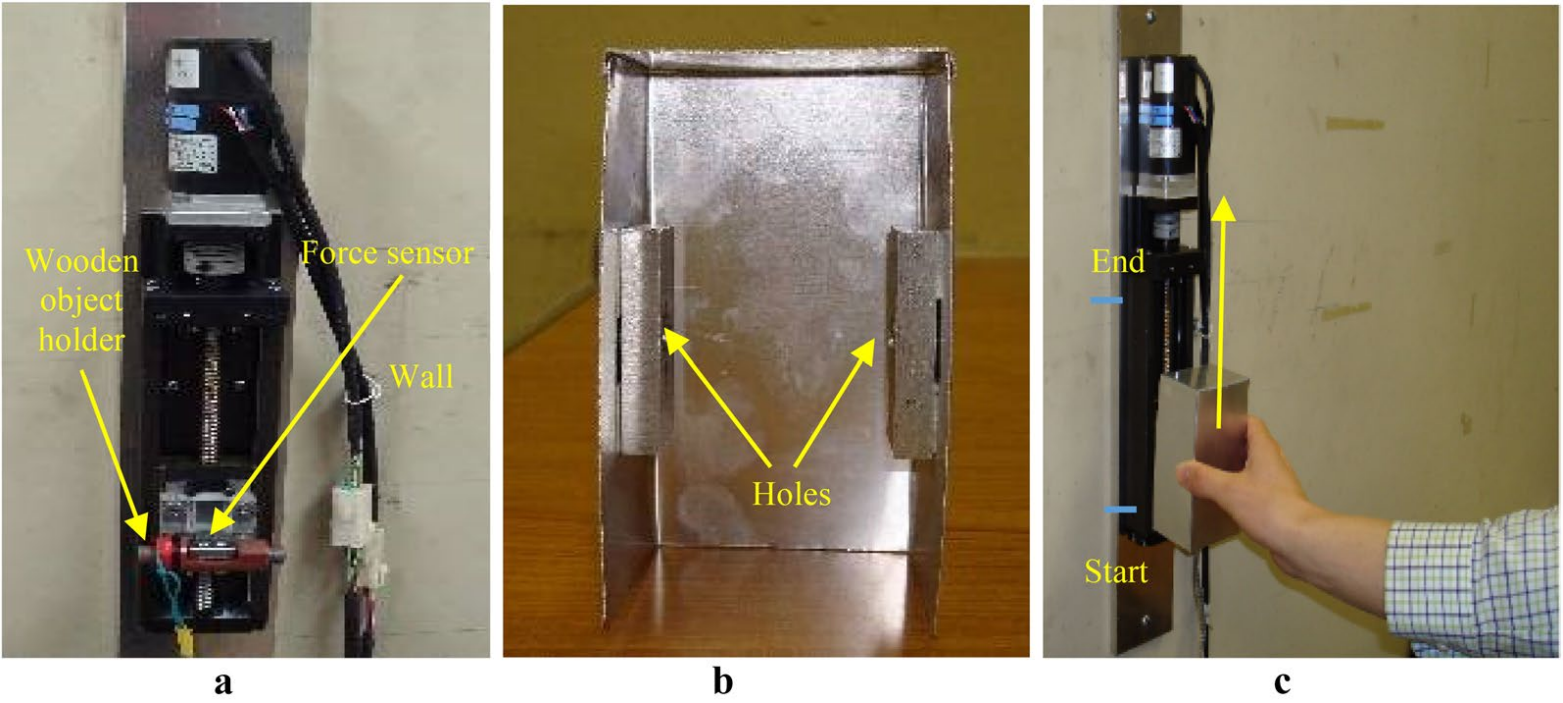
**a** 1-DOF PARS made of ball screw system for lifting lightweight objects, **b** an object (box), **c** a human user lifts an object with the PARS intuitively. In **c**, the marks show the initial (start) and the end/target position of the lifting trajectory

A simple 1-DOF PARS for lifting lightweight objects was developed as shown in Fig. 2. Figure 2a shows that an AC servomotor and a ball screw were fixed on a long rectangular metal plate coaxially, and then, the plate was vertically attached to the laboratory wall. A foil strain gauge-type force sensor was pasted on a small metal plate, and the plate was then attached to the ball nut of the ball screw system. A holder made of wood was connected to the force sensor plate. A rectangular box was made bending aluminum sheet (thickness 0.0005 m, dimensions 0.06 × 0.05 × 0.12 m, self-weight 0.016 kg). Two rectangular aluminum blocks with a hole in the center of each were attached inside the box to help tie the box to the holder (see Fig. 2b). This box was used as the object to be lifted with the PARS by a human user. The human user is to grip the object using a power grip and lift it (see Fig. 2c).

**Fig. 3:**
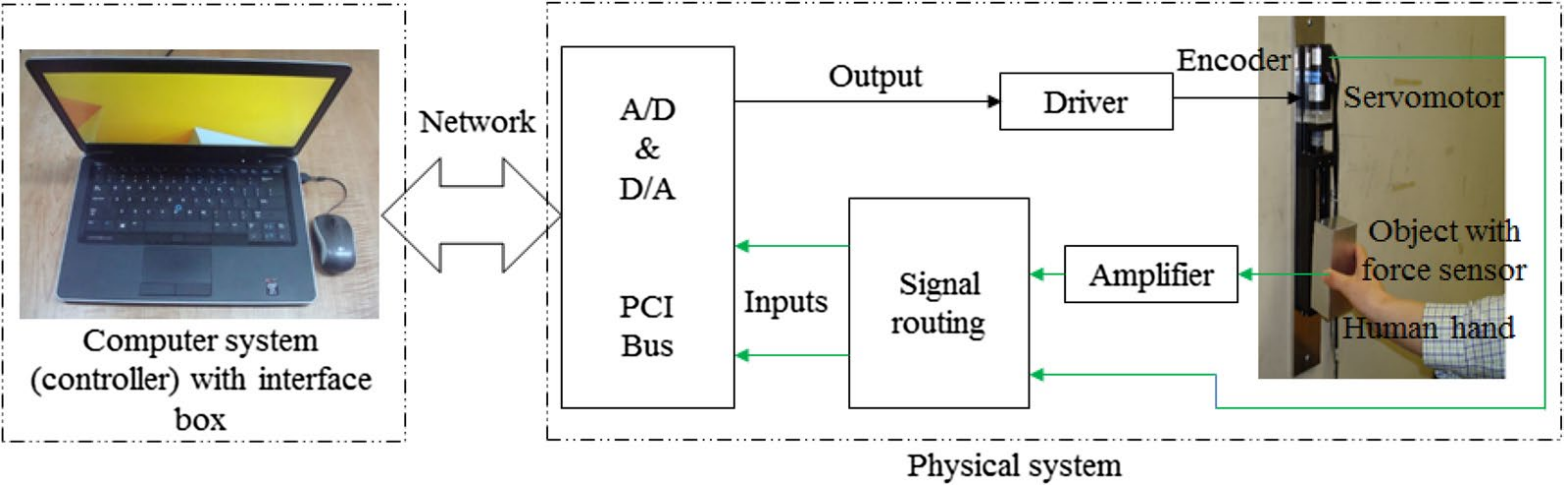
Experimental setup and system integration, major electronics components and the communication system for the PARS for lifting lightweight objects

Figure 3 depicts the experimental setup, system integration, system components and the communication. As shown in Fig. 3, the AC servo system primarily consisted of an AC servomotor with its controller, and the servo driver (servopack). The servo driver received a command signal in the form of a voltage signal from the control system (the controller in the computer) through a digital to analog (D/A) converter, amplified the signal and transmitted electric current to the servomotor that produced motion (acceleration) according to the commanded voltage. An encoder along with a counter attached to the servomotor measured object’s actual displacement and reported it to the servo driver. The servo driver compared actual displacement to the commanded displacement and corrected the commanded signal (pulse signal) if there was any error. The force applied to the object by the human was measured by the force sensor in the form of voltage signal, amplified by an amplifier and then sent to the control system via an analog-to-digital (A/D) converter. The force applied by the human user just contributed to generate motion of the lifted object.

## Weight perception-based dynamics model for manipulating objects with the PARS

In general, the trajectory for 6-DOF dexterous manipulation of objects with a PARS can be expressed by *X* as in (1), where *y*, *z* and *x* are the translational displacements and $\theta_{y}$, $\theta_{z}$ and $\theta_{x}$ are the rotational displacements along *y*, *z* and *x* axes, respectively, for the manipulation.

1 $$ X = \left[ {\begin{array}{*{20}c} y \\ z \\ x \\ {\theta_{y} } \\ {\theta_{z} } \\ {\theta_{x} } \\ \end{array} } \right] $$

In this article, as an initial initiative, we wanted to confine to the translational manipulation along the vertical direction to be performed using the PARS developed in “Design and Development of a simple PARS for lifting lightweight objects” section (see Fig. 2c). We assume that the *x*-axis indicates the vertical direction of manipulation. Hence, we consider the trajectory along the *x*-axis of (1) only. We emphasized manipulation along vertical direction because (1) the human feels the highest level of heaviness and thus may require the highest amount of power assistance for upward manipulation along vertical direction (against the gravitational weight of the object), and (2) such vertical lifting tasks are most common in industrial practices. However, the 6-DOF dexterous manipulation can be considered in the near future.

**Fig. 4:**
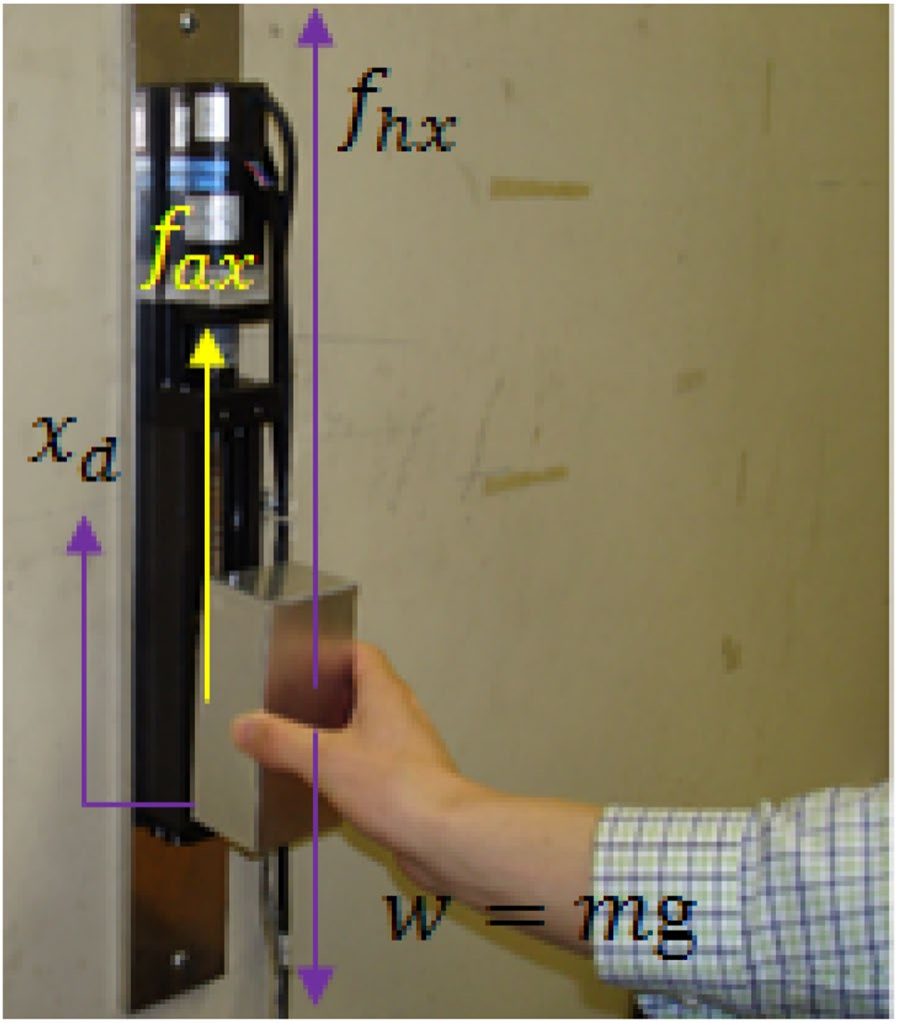
Dynamics for lifting an object by a human user with the PARS

Figure 4 shows that the dynamic behavior for lifting an object with the PARS in collaboration with a human user along vertical direction (along the *x*-axis) can be expressed in (2). Definition of each parameter in (2) is given in the list of symbols. To render the targeted free motion dynamics, the friction, viscosity, disturbances and actuating force can be ignored [38], and thus, the dynamics expression can be simplified to as in (3).

2 $$ m\ddot{x}_{d} + K_{x} \dot{x}_{d} + mg + F_{x} = f_{hx } + f_{ax} $$

3 $$ m\ddot{x}_{d} + mg = f_{hx} $$

We know from the characteristics of a PARS that the visually perceived weight of an object lifted with a PARS is different from the haptically perceived weight of the object [5]. To reflect such weight discrimination phenomenon in the dynamics, we adopted a special strategy based on the following hypothesis:

### **Hypothesis I**

The perception of object weight due to inertia might differ from the perception of weight due to gravity for manipulating an object with a power-assist robotic system.

To realize this hypothesis, we assumed that the mass parameter of the inertial force $(m\ddot{x}_{d} )$ in (3) would be different from that for the gravitational force (*mg*) [39], i.e., the dynamics expression in (3) might be modified to as in (4). In (4), *m*_1_ and *m*_2_ are the mass parameters of the inertial and the gravitational forces, respectively, and we considered $m_{1} \ne m_{2} .$ It is a novel approach of expressing (rendering) dynamics behavior for lifting objects with a PARS, where human’s cognitive features, i.e., weight perception/prediction, are taken into account because this behavior may be more helpful to the human operators/users regarding their cognitive skills [3, 19].

4 $$ m_{1} \ddot{x}_{d} + m_{2} g = f_{hx} $$

## Weight perception-based control system design for manipulating objects with the PARS

**Fig. 5:**
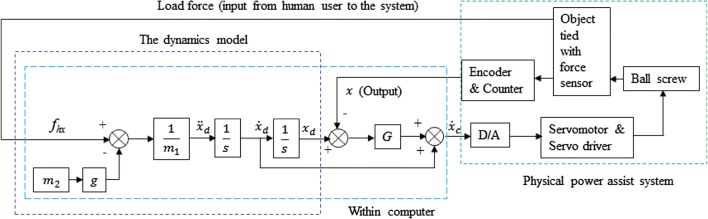
Weight perception-based admittance control with position feedback and velocity controller for the PARS

We designed a feedback position control scheme for the PARS for vertical lifting of objects using the weight perception-based dynamics expression in (4), which is shown in Fig. 5. The servo system was proposed to be kept on velocity control mode during implementation of the control system. In the control, the input was $f_{hx}$, and the output was *x* (and its derivatives). The relationship between $\dot{x}_{c}$ and the displacement error can be expressed in (5), where $\dot{x}_{c}$ is input to the servomotor through a D/A converter. The servomotor produced actuating force based on $\dot{x}_{c}$. As in Fig. 5, if $f_{hx} = 0$, it may be true that $f_{ax} \ne 0$. In this situation, the system may move the attached object upward or downward, but human intent is not to be reflected through $f_{hx}$, and thus, the system can not be treated as a human–robot system, and it does not provide expected flexibility [3]. In addition, the device may move involuntarily even if the operator does not apply any force (i.e., if $f_{hx} = 0$) due to incorrect inertia and gravity compensation. If so, this can be a very serious safety matter that does not comply with ISO15066 [37]. This is why we need to determine correct inertia and gravity compensation, which we will address later.

5 $$ \dot{x}_{c} = \dot{x}_{d} + G\left( {x_{d} - x} \right) $$

The control method shown in Fig. 5 falls within admittance control where input is the human force and output is the object displacement [12, 13]. The proposed control integrates positional feedback and velocity controller. For lifting objects, positional accuracy is very demanding that the admittance control can provide [12]. The position-based admittance control can also compensate inertia, friction, viscosity and other dynamic effects and nonlinear forces [29]. This is why we nominated the position-based admittance control for the PARS. The *g* is fixed, and hence, the human–robot system characteristics, HRI and the manipulation performance should depend upon mass values (*m*_1_ and *m*_2_), feedback gain (*G*) and the $f_{hx}$.We here call this proposed control scheme (Fig. 5) as the *Fixed Admittance Control Algorithm (FACA)* because the admittance parameters (*m*_1_, *m*_2_) remain fixed while the object is manipulated with the system.

As discussed earlier, selection of control strategies for power-assisted manipulation is challenging [29]. It is still an issue of argument about whether to use position-based controllers or torque/force-based controllers. It is still not widely decided about whether to use admittance controllers or impedance controllers. As above, we proposed feedback position controller with velocity controller in the form of an admittance controller. We also showed reasons behind such selection. The aim is to propose and establish a particular type of controller as the most rational controller for power-assist systems for object manipulation, and thus to end the unclear situations regarding controller selection for power-assisted object manipulation. The proposed control methods as above thus can contribute to the selection of appropriate controller and control parameters as well.

The presented FACA is a general admittance controller. But, the differences in mass parameters for inertia and gravity terms are totally novel. Such differences are also based on observed limitations of power-assist systems for object manipulation (i.e., mismatch between haptic and visual weight perceptions and related consequences), and such differences are adopted to remove or reduce the observed limitations. Such differences are based on a hypothesis, and such differences may impact HRI, system characteristics and performance. Hence, even though it may be easy to say that two mass parameters are used instead of one in the system dynamics and control, we believe that the impact and usefulness of such small consideration can be really very big. Again, none have thought in this different way, except us. Hence, we believe that it is novel and special. The proposed control seems to be simple, but it can be useful to solve the identified problems of PARSs for object manipulation. The control also possesses a set of novelties as follows: (1) It combines admittance control, feedback position control and velocity control, and (2) it has been made compatible with human users regarding their cognitive features, i.e., weight perception.

If we think of state-of-the-art advanced control theories or control engineering concepts, the presented controller approach may not be proven very complex and sophisticated. Our objective is to develop a simple controller, but it can serve greatly to solve the identified problems and improve the overall performance of the system for its intended applications. There are many complex controllers in the state-of-the-art literature, but only few controllers are actually proven effective in practical applications, and most of the proposed controllers are just theoretical analyses and are not proven in industrial applications especially in power-assisted manipulation. Hence, it is not an issue whether the proposed controller is complex or not. Instead, the issue is whether the proposed controller can solve the problems or not. In the proposed case, the simple controller is expected to be able to solve the problems and be proven effective. Furthermore, the proposed controller design considers human factors with control technology and augments the scope and applicability of the control theory. In addition, the control performance is planned to be evaluated with a comprehensive evaluation scheme. Such integration makes the concept novel and special, and worthwhile to be investigated, as follows.

## The control evaluation scheme

This section presents a novel comprehensive evaluation scheme that can be used to evaluate the proposed FACA (Fig. 5) for the PARS for manipulating objects. The evaluation criteria include HRI, system characteristics (kinematics and kinetics features) and manipulation performance. HRI is categorized into pHRI and cHRI, and the manipulation performance is expressed in terms of time efficiency and precision.

### pHRI assessment criteria and scale

**Fig. 6:**
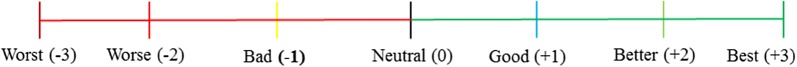
Bipolar and equal-interval rating scale with possible scores (in parentheses) for evaluating the pHRI

We propose a bipolar and equal-interval subjective rating scale (Fig. 6) [40] to assess the pHRI based on a few pHRI terms/criteria as described in Table 1.

**Table 1:** pHRI evaluation criteria with relevant description

pHRI criteria	Description of the criteria with respect to power-assisted object manipulation
Maneuverability ($M_{a}$)	Human’s haptic feelings while lifting an object with the PARS, e.g., perceived heaviness, kinesthetic and tactile perceptions, proprioception
Motion ($M_{o}$)	Nature of object’s velocity and acceleration felt by the human, e.g., whether the velocity and acceleration are low or high compared to the expectation of the human user
Naturalness ($N_{a}$)	Normalcy, convenience and likeability perceived by human user and non-complexity in operation while manipulating objects with the PARS
Stability ($S_{t}$)	Presence/absence of oscillations/vibrations, sudden inactivity of the system, etc., during manipulation and their effects on object manipulation, system structure and task environment
Health and safety ($H_{s}$)	Potential fatigue, injuries/accidents, impacts and jerks on human musculoskeletal system, etc.

In addition to basic health and safety assessment in Table 1, formal hazard analysis and risk assessment can be conducted for manipulating objects with the PARS [34–36]. Potential jerks and impact force on human musculoskeletal system and vibrations of the entire robotic system due to harmful manipulative motion at the time of object manipulation in collaboration between a human operator/user and the robotic system can be identified as the potential hazard. The risk can be assessed using (6), where $R_{j}$ is the risk of injury, $S_{j}$ is the severity of injury and $P_{o}$ is the probability of occurrence of the injury [35, 36]. Definition of the severity of injury with assigned subjective rating scores is presented in Table 2. The likelihood (probability) of occurrence of the hazard with the assigned rating score, and the definition of the level of risk for the specified range of risk are given in Table 3.

**Table 2:** Definition of the severity of injury with assigned subjective rating scores

Severity of injury	Description	Score
Catastrophic	System collapses (e.g., force sensor breaks, object is detached from the force sensor), user experiences little bleeding due to scratch or high impact on musculoskeletal system (hand, fingers), and the user needs to take primary medical care such as bandage, balm and pain killer	1.0
Severe	System does not break but stops with severe vibration, there is severe impact on musculoskeletal system, subject feels pain for a while, bleeding is about to occur, no medication is taken, but the subject experiences severe impact on hand and, for this reason, does not feel comfort to perform normal activities using hands for a few minutes after conducting the experiment	0.75
Moderate	Human experiences moderate jerks and impact on hand, but the human can tolerate it easily	0.50
Minor	Almost no jerk and impact on human hands, and the human has the same feeling as the human may experiences while lifting small objects in daily living, but still there is small jerk and impact	0.25

**Table 3:** Likelihood (probability) of occurrence with assigned rating scores, and definition of level of risk for specified range of risk

Likelihood (probability) of occurrence	Score	Range of risk	Level of risk
Frequent	1.0	$R_{j} >$ 0.6	High
Probable	0.8	$0.6 > R_{j} \ge 0.4$	Serious
Occasional	0.6	$0.4 > R_{j} \ge 0.2$	Medium
Remote	0.4	$R_{j} < 0.2$	Low
Improbable	0.2		

After a manipulation (lifting) trial, the experimenter based on his/her observation during the trial and on interviewing/asking the concerned user can determine the subjective rating score of severity of injury based on Table 2. This is the value of $S_{j}$. Definitely, some injuries need to occur. However, here injuries do not necessarily mean accidents or very harmful events. Even a small impact can be considered as an injury depending on the definitions in Table 2, but the severity can vary depending on the nature of the incident. $S_{j}$ needs to be available for each user at the end of the manipulation task. At the end of the manipulation by all users in a particular period, the probability (%) of occurrence of the injury can be calculated. For example, 20 users separately use the system to lift an object in 20 trials. We assume that it is found that the catastrophic injury (based on the definitions in Table 2) occurs in 3 trials out of 20 trials. Then, the likelihood or probability of catastrophic injury is 3/20 = 0.15, and this is the $P_{o}$ value for catastrophic injury in general. Such general $P_{o}$ values for other types of injuries such as severe, moderate and minor need to be calculated. Now, we give an example of how $R_{j}$ can be calculated for a user for a lifting trial following (6). Say, after a trial, the $S_{j}$ value is determined as 1, i.e., $S_{j} = 1$, which indicates catastrophic injury. The general likelihood or probability of catastrophic injury is 0.15, i.e., $P_{o} = 0.15$. Hence, the $R_{j}$ is 0.15 according to (6) [33–36]. The explanation as above shows that the overall risk depends on both severity of injury and probability of occurrence. It means that the risk may be low even if a catastrophic injury occurs very less frequently, but the risk may be high even if a moderate/minor injury occurs very frequently.

6 $$ R_{j} = S_{j} \times P_{o} $$

### cHRI assessment methods and metrics

**Fig. 7:**
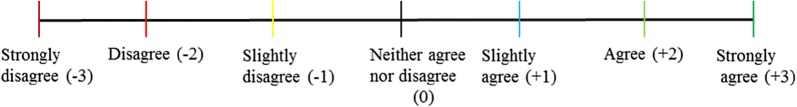
Likert scale with possible scores in parentheses to assess human user’s trust in the PARS

The cHRI can be expressed in a few terms such as cognitive workload of the human user and the user’s trust in the PARS. NASA TLX can be used to assess the workload, which is expressed in 6 dimensions such as mental demand, physical demand, temporal demand, performance, frustration and effort [41]. Trust is the willingness of the user to rely on or to believe in the assistance provided by the PARS [20, 21]. Trust can be assessed using a Likert scale [42] as shown in Fig. 7.

### Evaluation of human–robot manipulation performance

Human–robot co-manipulation performance can be expressed in two terms: precision and time efficiency. Deviation from target position can be used to measure the precision objectively using (7), where $P_{m}$ is the measured position and $P_{t}$ is the target position. Time efficiency is the ratio between targeted co-manipulation time ($T_{t}$) and measured co-manipulation time ($T_{m}$) as in (8).

7 $$ {\text{Precision}} = \left( {1 - \frac{{|P_{t} - P_{m} | }}{{P_{t} }}} \right) \times 100\% $$

8 $$ {\text{Time}}\;{\text{Efficiency}} = \left( {\frac{{T_{t} }}{{T_{m} }}} \right) \times 100\% $$

The proposed evaluation scheme as above includes assessments of pHRI and cHRI. The pHRI assessment also includes risk assessment and analysis. Risk analysis can confirm adhering to the guidance of ISO/TS 15066, ISO 10218-1 and ISO 10218-2 [37]. The overall manipulation performance of the PARS such as the efficiency and the precision is also included in the evaluation scheme. All these can make the evaluation scheme comprehensive.

We used HRI as evaluation criteria because power-assist system is a human/user interactive system, and thus, evaluation of HRI is essential to achieve human/user-friendliness in collaborative manipulation from both physical and cognitive points of views. System characteristics are the own characteristics of the power-assist system. These criteria are necessary to understand and evaluate the performance of the system itself. Manipulation performance is related to the objective of the power-assist system. Precision indicates the quality of manipulation. It may be also related to safety because unprecise manipulation, i.e., manipulation to a wrong or undesired location, may hit the user and be unsafe. Efficiency is related to manipulation productivity, which is very important for any industrial operation including material manipulation or transfer. In fact, efficiency and precision are the key performance indicators (KPIs) of power-assisted or other types of object manipulation in actual industrial applications. This is why we used manipulation performance especially efficiency and precision as evaluation criteria. HRI, system characteristics and manipulation performance are also interrelated, i.e., HRI depends on these criteria and the vice versa. For example, if load force is very high, acceleration will also be high, and thus, the manipulation may be unsafe and the maneuverability may go down. We do not claim that these are the only evaluation criteria. However, these criteria can cover most of the criteria that users may expect from a power-assist device in industrial applications.

## Experiment 1: Evaluation of the FACA for lifting lightweight objects

### Objective of the experiment

The objective of this experiment was to evaluate the FACA (Fig. 5) for the PARS (Fig. 2) for lifting lightweight objects for various control parameters (*m*_1_ and *m*_2_ values) using the evaluation scheme introduced in “The control evaluation scheme” section.

### Control requirements

The control requirements were to produce optimum (satisfactory) HRI and manipulation performance.

### Recruitment of subjects

Sixty subjects (engineering students) were recruited and randomly divided into three groups (Group I 16 males, 4 females, mean age = 23.47 years, STD = 2.83; Group II 18 males, 2 females, mean age = 24.52 years, STD = 2.31; Group III 20 males, mean age = 25.89 years, STD = 3.11). The purpose of dividing the subjects into several groups was to use the subjects for different experiment protocols as presented later. The subjects did not report any physical and mental problems regarding their health. The subjects supported the experiments voluntarily. The study was approved by the concerned ethical committee (equivalent to institutional review board, IRB).

### Design of experiment

In total, we selected 36 pairs of *m*_1_ and *m*_2_ values (Table 4) as the first guess based on our experience. We did not select *m*_1_ = 0 and *m*_2_ = 0 because zero inertia (*m*_1_ = 0) might produce oscillations and instability, and the human might lose haptic senses partly at zero gravity (*m*_2_ = 0), which might result in poor HRI and performance [22]. The independent variables were *m*_1_ and *m*_2_ values, and the dependent variables were: (1) HRI (pHRI and cHRI) including the risk, (2) system characteristics (kinetics–load force, kinematics–displacement, velocity, acceleration) and (3) manipulation performance (precision, time efficiency).

**Table 4:** Values of *m*_1_ and *m*_2_ (6 × 6 pairs) used in the experiment

m_1_ (kg)	0.25	0.50	0.75	1.0	1.25	1.5
*m*_2_ (kg)	0.25	0.50	0.75	1.0	1.25	1.5

### Experimental procedures and data records

Only Group I subjects were asked to participate in this experiment. We provided detailed instructions to the subjects about the experimental methods and the evaluation scheme. Each subject participated in the experiment separately. The FACA for the PARS in Fig. 5 was implemented using MATLAB/Simulink as specified in Table 5. In each trial, a pair of values of *m*_1_ and *m*_2_ from Table 4 was randomly selected and put in the control system, and its confidentiality was maintained. Then, a subject was asked to perceive the weight of the object visually before touching the object, and the subject’s response was recorded properly. Then, the subject lifted the object with the PARS up to a targeted height of 0.1 m ($P_{t}$ in (7)) and then released the object. Figure 2c shows the detailed procedures. To make the subject aware of the initial and target positions, we marked the initial and target positions using a marker pen/colored tape during the experiment (see Fig. 2c). For each pair of values of *m*_1_ and *m*_2_, we asked the subject to repeat the trial for three times and also instructed the subject to perform three consecutive lifts (including the arrangement time between two consecutive lifts) within 9 s (hence, the targeted time for a trial, $T_{\text{t}}$ in (8) was 3 s). To make the subject aware of the target time, we put an online timer displayed in a monitor in front of the subject. The timer started at the target time duration (e.g., 9 s) and then reduced to zero (0 s) gradually. An assistant to the experimenter gave a warning sound 2 s before the timer reached zero and asked the subject to stop when the timer displayed zero. The timer and warnings worked as visual and auditory feedbacks to the subject, respectively. We recorded the system characteristics separately after each trial. At the end of the three trials, the subject evaluated the pHRI and the cHRI once following the evaluation scheme in “The control evaluation scheme” section. We recorded the total time required for the three consecutive lifts for the pair of values of *m*_1_ and *m*_2_ using a stopwatch. In the same procedures, we conducted the experiment for each of the 36 pairs of *m*_1_ and *m*_2_ for each of the 20 subjects separately. We used $G = - 5.75$ and determined it by trial and error. We also calculated $R_{j}$ for each trial based on the $R_{j}$ assessment method of “pHRI Assessment Criteria and Scale” section.

**Table 5:** Details of the Simulink environment

Criteria	Specifications
Solver name	ode4 (fourth-order ordinary differential equation)
Solver method	Fourth-order Runge–Kutta
Solver type	Fixed time step
Sample time	0.001 s

**Table 6:** Mean (*n *= 20) PLF, peak acceleration and peak velocity with standard deviations in the parentheses for the first and the third (last) lifts of the subjects for *m*_1_ = 0.5 kg, *m*_2_ = 0.25 kg

Lift (trial)	PLF (N)	Peak acceleration (m/s^2^)	Peak velocity (m/s)
First lifts	7.41 (0.89)	1.78 (0.11)	0.47 (0.09)
Third lifts	7.32 (0.66)	1.74 (0.12)	0.45 (0.07)

#### *Remark 1*

The just noticeable difference (JND) or difference limen (DL) is the difference in stimuli that the subject notices some proportion *p*^1^ of the time (50% is usually used for *p*^1^). In the branch of experimental psychology focused on sense, sensation and perception, which is called psychophysics, a JND is the amount something must be changed in order for a difference to be noticeable and detectable at least half the time [43]. Weber found that a weight seems to have to increase by 5% for someone to be able to reliably detect the increase, and this minimum required fractional increase (of 5/100 of the original weight) is referred to as the “Weber fraction” for detecting changes in weight [44, 45]. Hence, the JND for weight perception was 5%. In Table 4, there was a minimum change of 0.25 kg in each experiment, which is larger than 5%. Again, the weight change was random because in each trial a pair of values of *m*_1_ and *m*_2_ from Table 4 was randomly selected and put in the control system, and its confidentiality was maintained. Hence, each trial was independent, random and there was also time delay between two trials. As a result, no question can arise regarding the JND perceived by the subjects [44, 45].

## Results of experiment 1 with analyses

We determined the mean (*n *= 20) visually perceived weight of the object, which was 2.48 kg.

### Evaluation of the pHRI

We developed a set of objective functions as in (9) and (10) and an optimization algorithm (Algorithm 1) to determine the *m*_1_ and *m*_2_ pair(s) that might produce optimum pHRI for two sets of constraints (the hard constraints that were required to be satisfied and the soft constraints that were desired to be satisfied). We decided the constraints based on our experiences. In (9), *i* is the subject, i.e., *i *= 1, 2, 3, …, 20, and $C_{1} , C_{2} , \ldots C_{5}$ are the positive-valued constants that indicate relative importance (weight) of the evaluation criteria. For simplicity, we used $C_{1} = C_{2} = C_{3} = C_{4} = C_{5} = 1$, i.e., all the evaluation criteria carried equal weight. However, the values of the weights can vary depending on the importance of the criteria that can be assessed using a subjective rating scale (Fig. 7). System identification methods such as autoregressive moving average model (ARMAV) [46] might also be used to identify the values of the weights. The *m*_1_, *m*_2_ pair with the highest $\varvec{J}_{1} \varvec{ }$ value and the lowest $\varvec{J}_{2}$ value that passed the constraints would be considered as the *m*_1_, *m*_2_ pair producing the optimum pHRI.

The optimization results showed that no $J_{1}$ and $J_{2}$ values for the *m*_1_, *m*_2_ pairs returned for the soft constraints. However, for the hard constraints, for $m_{1} = 0.5\,{\text{kg}},m_{2} = 0.25\,{\text{kg}}$, $J_{1}$ had the highest value and $J_{2}$ had the lowest value, and thus, *m*_1_ = 0.5 kg, *m*_2_ = 0.25 kg were considered as the admittance control parameters producing the optimum pHRI.
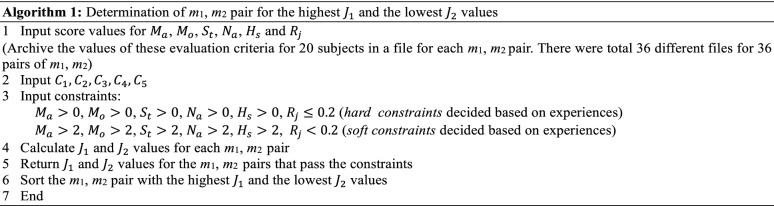

9 $$ J_{1} \left( {m_{1} , m_{2} } \right) = C_{1} \mathop \sum \limits_{i = 1}^{20} M_{a} + C_{2} \mathop \sum \limits_{i = 1}^{20} M_{o} + C_{3} \mathop \sum \limits_{i = 1}^{20} S_{t} + C_{4} \mathop \sum \limits_{i = 1}^{20} N_{a} + C_{5} \mathop \sum \limits_{i = 1}^{20} H_{s} $$

10 $$ J_{2} \left( {m_{1} , m_{2} } \right) = \mathop \sum \limits_{i = 1}^{20} R_{j} $$

### Evaluation of the cHRI

We developed a set of objective functions as in (11) and (12) and an optimization algorithm (Algorithm 2) to determine the *m*_1_ and *m*_2_ pair(s) that might produce optimum cHRI for the hard and soft sets of constraints, where $W_{t}$ was the total cognitive workload and $T_{l}$ was the trust level. The *m*_1_, *m*_2_ pair with the lowest $J_{3}$ value and the highest $J_{4}$ value that passed the constraints would be considered as the *m*_1_, *m*_2_ pair producing the optimum cHRI. Results showed that no $J_{3}$ and $J_{4}$ values for the *m*_1_, *m*_2_ pairs returned for the soft constraints. For the hard constraints, for $m_{1} = 0.5\,{\text{kg}},m_{2} = 0.25\,{\text{kg}}$, $J_{3}$ had the lowest value and $J_{4}$ had the highest value. Thus, *m*_1_ = 0.5 kg, *m*_2_ = 0.25 kg were considered as the control parameters producing optimum cHRI.
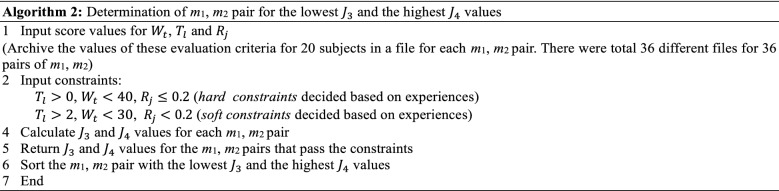

11 $$ J_{3} \left( {m_{1} , m_{2} } \right) = \mathop \sum \limits_{i = 1}^{20} W_{t} $$

12 $$ J_{4} \left( {m_{1} , m_{2} } \right) = \mathop \sum \limits_{i = 1}^{20} T_{l} $$

As above, *m*_1_ = 0.5 kg, *m*_2_ = 0.25 kg were found as the control parameters producing optimum pHRI (through Algorithm 1) and cHRI (through Algorithm 2). Hence, *m*_1_ = 0.5 kg, *m*_2_ = 0.25 kg were considered as the control parameters producing optimum HRI (pHRI + cHRI). The results also thus justify Hypothesis I that the novel strategy of consideration of difference in the mass parameters between the inertial and the gravitational forces for determining dynamics of human–robot collaborative manipulation can help achieve optimum HRI [47]. This is also the implication of selecting *m*_2_ (the virtual gravity) different from the actual gravity and from the inertial mass (*m*_1_). The opposite is also true, i.e., we cannot achieve optimum or satisfactory HRI if we do not reflect weight perception in manipulation dynamics and control [47].

Analysis of variance (ANOVA) conducted on the evaluation scores for the 5 pHRI criteria for the 20 subjects for *m*_1_ = 0.5 kg, *m*_2_ = 0.25 kg showed that variations in pHRI between the subjects were not statistically significant for each of the pHRI criteria (e.g., for motion, $$ F = 3.61, $$ *p *> 0.05), which can indicate the generality of the results. Similarly, variations in cHRI scores between the subjects were not statistically significant (*p *> 0.05 at each case). We believe that the PARS produced optimum HRI for hard constraints, which was possible due to consideration of weight perception in the derivation of dynamics and in the design of the control [22], but further efforts might be helpful to achieve optimum HRI for the soft constraints.

#### *Remark 2*

Here, same *m*_1_ and *m*_2_ values (*m*_1_ = 0.5 kg, *m*_2_ = 0.25 kg) were found as the control parameters for producing both optimum pHRI (through Algorithm 1) and cHRI (through Algorithm 2). Hence, it was easy to consider *m*_1_ = 0.5 kg, *m*_2_ = 0.25 kg as the control parameters producing optimum HRI (pHRI + cHRI). However, if the optimization results for pHRI and cHRI confer upon different pairs of *m*_1_ and *m*_2_, then the *m*_1_ and *m*_2_ pair with the lower risk ($R_{j}$) can be considered as the control parameters producing optimum HRI.

#### *Remark 3*

The proposed optimization method falls within the “local optimization” concept, and it is an exhaustive search method/tool [48]. This method is in line with the classical optimization method named “single variable optimization” [47]. We here search the optimum condition (*m*_1_ and *m*_2_ pair) through this optimization algorithm. Here, the optimum *m*_1_ and *m*_2_ pair can also be termed as the best or the most feasible *m*_1_ and *m*_2_ pair. However, use of more *m*_1_ and *m*_2_ values (1.5 > *m*_1_ > 0 and 1.5 > *m*_2_ > 0) as well as large control gains (*G*) might be an alternative approach to search for more general/global optimization results for the pHRI though it could be exhaustive, and large gains might also reduce the performance [48].

The proposed HRI optimization is simple, but it seems to be useful and beneficial to achieve optimum/best HRI for PARSs for object manipulation. We would not be able to sort out the optimum/best *m*_1_ and *m*_2_ pair out of many (here, 36) without using such an optimization method. The proposed optimization approach is novel as such approach is usually not applied to the state-of-the-art PARSs for object manipulation [6–17]. However, the novelty is not in the optimization theory, but in the application. Even though the proposed optimization is a general well-known optimization formula, it was necessary to formulate the optimization problem, identify the evaluation parameters and weights, measure the parameters and weights, and determine the thresholds for this particular application, which are not trivial. Thus, this is definitely a contribution and a novelty that can augment the application paradigm of optimization theories as well as can benefit the PARSs to be suitable and human-friendly for industrial applications.

### Evaluation of the system characteristics

**Fig. 8:**
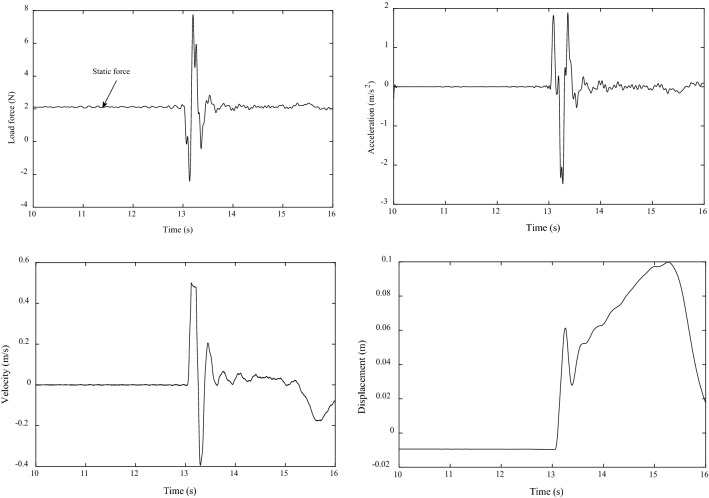
Typical kinetics (load force) and kinematics (acceleration, velocity, displacement) data for a subject for lifting a lightweight object with the PARS for the first lift of the three consecutive lifts with *m*_1_ = 0.5 kg, *m*_2_ = 0.25 kg used in the control system

Figure 8 shows the typical system characteristics, i.e., the kinetics (load force) and the kinematics (acceleration, velocity, displacement), for a subject for lifting an object with the PARS for *m*_1_ = 0.5 kg, *m*_2_ = 0.25 kg used in the control system. The actually required load force to lift an object comfortably is usually slightly larger than the object’s simulated weight (static force) [18, 19]. We found *m*_2_ = 0.25 kg as the optimum condition and used it in the control system. Hence, the simulated weight of the object was *m*_2_*g *= 0.25 × 9.81 N = 2.45 N. This is the static force. The load (lifting) force should be slightly larger than this force. This is the requirement for lifting an object in terms of load force [18]. Figure 8 shows that the static force is around 2.45 N, and the load force beyond this amount is excessive that can cause excessive acceleration. We compare the load force profile of Fig. 8 to this standard, and it appears that the peak load force (PLF) was approximately 3 times larger than the actual requirement. We assume that the peak acceleration was also large/excessive as acceleration is usually proportional to load force [18].

### Learning effects on the system characteristics

Table 6 shows the mean PLF, peak acceleration and peak velocity for the first and the third lifts (trials) of the subjects for *m*_1_ = 0.5 kg, *m*_2_ = 0.25 kg. The results in Table 6 show that the PLF, peak acceleration and peak velocity for the third lifts reduced as the subjects gained some experience and learned through three repeated manipulation. However, the reductions in PLF, peak acceleration and peak velocity were small, which indicate that the effects of weight illusion were not mitigated even after the human had gained experience when manipulating objects with the PARS. We conducted ANOVAs separately on the measured PLF, peak acceleration and peak velocity for the first and the third lifts for the 20 subjects for *m*_1_ = 0.5 kg, *m*_2_ = 0.25 kg, which showed that the variations in PLF, peak acceleration and peak velocity between the lifts and between the subjects were not statistically significant (e.g., for the PLF, between the first and the third lifts, $$ F = 1.33, $$ *p *> 0.05; between the subjects, $$ F = 3.57, $$ *p *> 0.05). Thus, the results can be treated as general results. The results also proved the necessity of the proposed weight perception-based feed-forward control of load force for power-assisted manipulation [7, 8].

## Novel variable inertia admittance control

Figure 8 shows that the PLF and peak acceleration were larger than the actual requirements as we mentioned earlier [18]. We believe that the PLF and peak acceleration could further increase if weight perception was not included in the control system, i.e., *m*_1_ = 0.5 kg, *m*_2_ = 0.25 kg were not used as the optimum/best control parameters. We further believe that the large PLF and peak acceleration hindered achieving optimum HRI for the soft constraints (see Algorithms 1 and 2). Thus, it is realized that the HRI could be further improved (i.e., the optimum HRI could be obtained for soft constraints as well) if an active compliance control method in the form of a variable admittance control was applied to reduce excessive PLF and peak acceleration [13].

**Fig. 9:**
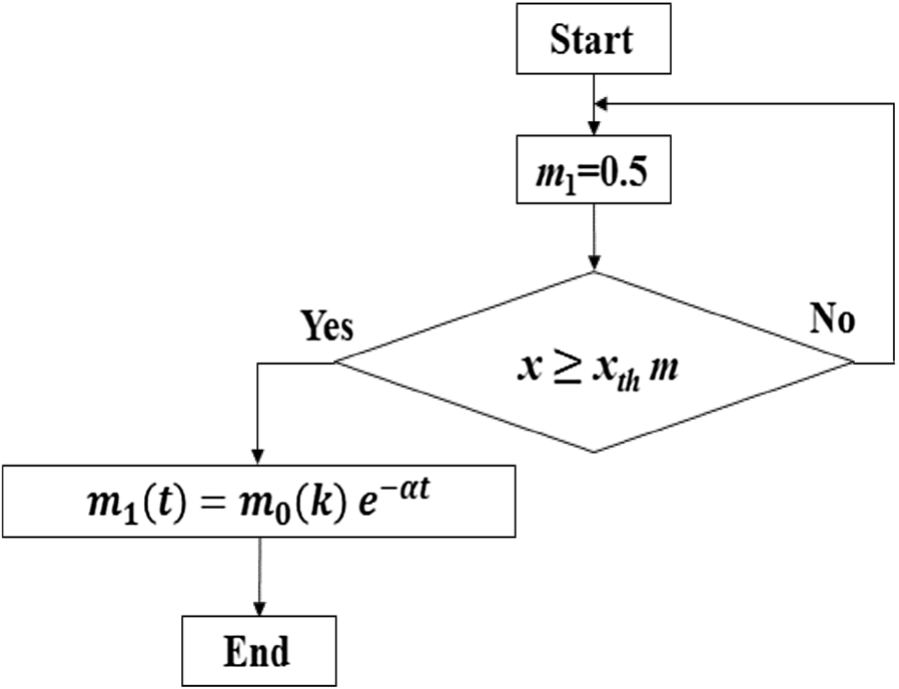
Novel *Variable Admittance Control Algorithm (VACA)* for the PARS for manipulating (lifting) objects

It was proved in [19] that *m*_1_ does not influence weight perception, but it influences kinetics (load force) and kinematics (acceleration) for power-assisted object manipulation. However, *m*_2_ affects weight perception, kinetics and kinematics [19]. Hence, the virtual mass *m*_1_ can be varied to adjust the acceleration and precision in power-assisted manipulation [13]. The novel active compliance control can be formulated in such a way that the value of *m*_1_ exponentially declines from a large value (say, *m*_1_ = 2.0 kg) to a small value (say, *m*_1_ = 0.5 kg), while the human lifts an object with the PARS and the vertical displacement exceeds a pre-specified threshold. Declination in *m*_1_ can reduce the PLF (human input) and the resulting acceleration proportionally, but may not affect human’s haptic perceptions through the relationship in (3) because a human does not feel the change of *m*_1_ [19]. Such a control concept can be modeled as an exponential decay function of *m*_1_ as in (13), where *k* is the time when the exponential decay starts, *t* is the time when the decay ends, *m*_0_ is the value of *m*_1_ when the decay starts at *k*, *m*_1_ = 0.50 kg when the decay ends at *t*, and *α* is the decay constant. We may consider $T = t - k$ as the duration of the exponential decay. The novel control based on this concept is shown in Fig. 9, where *x*_th_ is a displacement threshold. In order to modulate the control for specific requirements or to address various situations on dynamic contexts, we can adjust the proposed variable admittance control in several ways as follows:

1. Exponential decays can be arranged consecutively for *n* times (*n* is a positive real number) during a single lifting trial,
2. The values of *α*, $m_{0}$, *k*, *t* and *T* can be modulated.

13 $$ m_{1} \left( t \right) = m_{0} \left( k \right) e^{ - \alpha t} $$

This control approach can be treated as a *Variable Admittance Control Algorithm (VACA)* because the inertia mass (*m*_1_) varies with time. The algorithm is novel in the senses that (1) unlike the state-of-the-art practice [13], only the inertia mass (*m*_1_) varies instead of the gravity mass (*m*_2_), which can help change (reduce) the PLF and peak acceleration without changing haptic perceptions (such changes may adversely affect the HRI), and (2) optimum *m*_1_ and *m*_2_ values are used. The proposed control is practical as the exponential decay is triggered by a position threshold instead of an instantaneous time threshold. The exponential decay of *m*_1_ needs to start when/before the peak load force is the maximum. We know from the literature that the peak load force is the maximum when the subject just starts lifting at the very initial phase of lifting [18]. The position threshold can successfully indicate such initial phase and can be very general. In contrast, if we use a time-based threshold, e.g., if the control strategy arranges an exponential decay of *m*_1_ starting at a particular time, then generalization of this particular time is difficult. The proposed position-based threshold approach is an empirical ad hoc approach that may also require additional effort to tune in the case of a different target pose. But, in all lifting trials, the object must cross/pass the initial phase of lifting along its trajectory (here, 0.01 m for lightweight object). The position-based condition $x \ge x_{\text{th}}$ as Fig. 9 shows can cover a wide range of target poses. Even though the position/displacement-based thresholds may need to be adjusted if the targeted manipulation trajectory length increases/decreases significantly, it is easy to adjust such situations.

We can customize the proposed VACA for our case. We see in Fig. 8 that for lifting a lightweight object with a PARS, the PLF and the peak acceleration are synchronized. The load force and acceleration reach the peak toward the positive direction just after 13 s when the object just starts to move up, and end before 14 s, i.e., the PLF and peak acceleration last for the first 1 s of the lift duration only. Hence, the control strategy arranges an exponential decay of *m*_1_ starting at when the object just starts to move up (e.g., when $x_{\text{th}} = 0.01\,{\text{m}}$ at corresponding *k* $\approx$ 13.1 s) and ending after 1 s (the corresponding *t* may be 14.1 s) making *T *= 1 s. We may assume *m*_0_ = 1.5 kg (a large value of *m*_1_ at the initial stage of lifting at 13.1 s), and it then declines to *m*_1_ = 0.5 kg within 1 s (at around 14.1 s). We determined the decay constant (*α*) following (13), which was |1.03| s^−1^. The empirically estimated VACA parameter values for lifting a lightweight object with the PARS are given in Table 7 (see VACA (light) in Table 7). Table 7 also presents such values in case a heavy object is manipulated with a suitable PARS, which is presented later. Note that we may consider these values as the general findings for lifting objects with a PARS.

**Table 7:** Empirically decided VACA parameters for lifting light and heavy objects with the PARS

Control strategy	VACA parameters					
	*m*_0_ (kg)	*x*_th_ (m)	*k* (s)	*t* (s)	*T* (s)	*α* (s^−1^)
VACA (light)	1.5	0.01	13.1	14.1	1.0	1.03
VACA (heavy)	10	0.05	0.5	1.5	1.0	3.1

## Experiment 2: Verification and validation of the VACA for lifting lightweight and heavy objects

### Experimental objectives

The objectives of this experiment were: (1) to verify the effectiveness of the VACA in improving HRI and manipulation performance for lifting lightweight objects and then (2) to validate the VACA for lifting heavy objects with power-assist. To address the first objective, we compared the evaluation results between the FACA and the VACA for lifting a lightweight object using the experimental system shown in Fig. 2. To address the second objective, we compared the evaluation results for the VACA for lifting a lightweight object with the PARS (Fig. 2) to that for lifting a heavy object with another suitable PARS.

### Hypothesis

We adopted the following hypothesis for experiment 2:

#### **Hypothesis II**

The VACA can be made effective to produce similar HRI and manipulation performance for power-assisted manipulation of both lightweight and heavy objects.

### Development of the PARS for manipulating heavy object

**Fig. 10:**
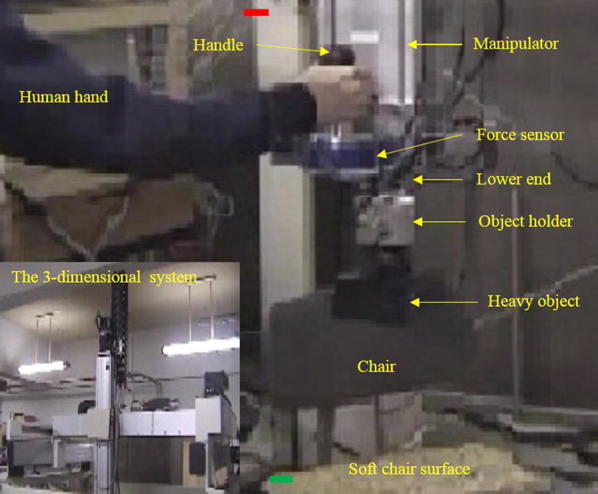
Human vertically lifts a heavy object with the PARS. The whole system is shown in the lower left corner. The green and red marks tentatively show the initial and target positions of the object in a lifting trial, respectively

We developed a PARS as shown in Fig. 10 for manipulating heavy objects. The system consisted of a vertical linear (Cartesian) manipulator. A force sensor was attached to a handle, and then, it was attached to the manipulator near its lower end. A heavy object was tied to an object holding device (object holder), which was then tied to the manipulator at its bottom (lower end). The human applied grip and load forces at the handle using a power grip while lifting the heavy object with power-assist. The object was kept on a soft chair top before it was lifted by the human.

### Verification of the FACA and the VACA for lifting lightweight objects using step voltage responses

**Fig. 11:**
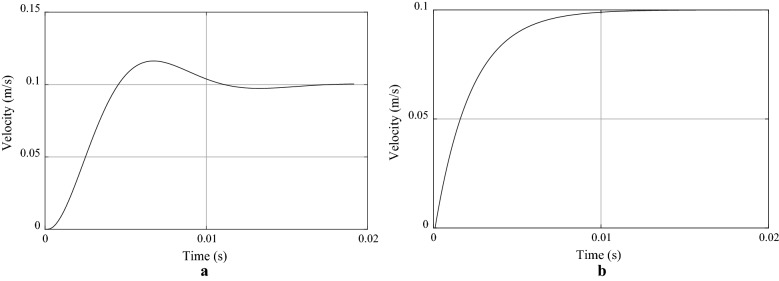
Step voltage responses (in term of velocity) of the PARS for lifting a lightweight object for **a** the FACA and **b** the VACA

Before experimental validation of the novel VACA with heavy object, we at first verified the effectiveness of the VACA through step voltage responses in an auxiliary experiment. To do so, we applied a step voltage command to the PARS (Fig. 2) for lifting a lightweight object for the FACA and the VACA separately. The responses are shown in Fig. 11, which indicate that the system generated slight overshoot for the FACA, but no overshoot was generated for the VACA. The responses were fast for both cases. We believe that the slight overshoot for the FACA restricted the assist system producing optimum HRI and performance for the soft constraints in experiment 1. We further believe that the VACA removed the overshoot by adding active compliance to the assist system, which indicates the potential effectiveness of the VACA.

Effectiveness of the control algorithms could be further justified through closed-loop control analysis for the system. For example, a ramp response might show tracking error between the actual and the reference trajectories as well as the stability of the system in terms of oscillations, and a Bode plot might show the system bandwidth and stability [49]. However, we used experimental validation approaches to address these issues as well as to validate the control algorithms using heavy objects as follows. We estimated the optimum control parameters in experiment 1 using the experimental approach instead of system identification methods [50]. We followed experimental validation approaches because evaluation of control systems based on experimental results might be more practical and reliable. However, the verification and analysis in Fig. 11 based on step responses can forecast some implications of potential effectiveness of the system before we validate the system using heavy objects. Thus, the step voltage responses are to be complementary with the experimental validation results.

### Experimental procedures

We implemented the VACA for two experiment protocols (control strategies) separately, as follows:

1. *VACA with lightweight object*: In this protocol, the control system in Fig. 5 was implemented for lifting a lightweight object with the PARS shown in Fig. 2 using the control parameters *m*_1_ = 0.5 kg, *m*_2_ = 0.25 kg only. The *m*_1_ value was also modified in real time based on the VACA in Fig. 9. Only Group II subjects were asked to separately participate in this experimental protocol. We used the empirically determined VACA parameters for the lightweight object given in Table 7. In addition, we used $P_{\text{t}} = 0.1\,{\text{m}}$, and $T_{\text{t}} = 3\,{\text{s}}$.
2. *VACA with heavy object*: In this protocol, the control system in Fig. 5 was implemented for lifting a heavy object (about 7.5 kg) with the power-assist device shown in Fig. 10 using control parameters *m*_1_ = 0.5 kg, *m*_2_ = 0.25 kg only. The *m*_1_ value was also modified in real time based on the VACA shown in Fig. 9. Only Group III subjects were asked to separately participate in this experimental protocol. We used the empirically determined VACA parameters for heavy objects given in Table 7. We also used $P_{\text{t}} = 0.5\,{\text{m}}$ and $T_{\text{t}} = 15\,{\text{s}}$.

For both protocols in experiment 2 as above, the evaluation scheme, control requirements, experimental design and experimental procedures, and data records were same as those applied to experiment 1 in “Experiment 1: evaluation of the FACA for lifting lightweight objects” section.

## Results of experiment 2

Experiment 1 for lifting lightweight object using the system in Fig. 2 based on the control system in Fig. 5 for only *m*_1_ = 0.5 kg, *m*_2_ = 0.25 kg as control parameters is called here the FACA with light object. This section compares the evaluation results among (1) FACA with light object (experiment 1), (2) VACA with light object (Fig. 2, experiment 2) and (3) VACA with heavy object (Fig. 10, experiment 2) to justify the effectiveness of the VACA for lifting heavy objects.

### Improvement in system kinematics and kinetics

Table 8 shows that the PLF and peak acceleration reduced significantly due to the VACA for both lightweight and heavy objects (experiment 2) in comparison with those for the FACA (experiment 1). The results thus indicate the effectiveness of the novel VACA. We see that the PLF, acceleration and velocity were slightly larger for the heavy object for the VACA. It happened because the large size and more heaviness influenced the subjects to apply larger input load force while manipulating object [18]. Larger input force also generated larger velocity and acceleration [18]. In our case, the mean visually perceived weight of the heavy object (Fig. 10) in experiment 2 was 7.62 kg, which was about three times larger than the visually perceived weight of the light object (Fig. 2) in experiment 1 (2.48 kg). Hence, the PLF, velocity and acceleration for the heavy object were supposed to be three times larger than that for the lightweight object [18]. However, it did not happen. In fact, the differences in PLF, velocity and acceleration between lightweight and heavy objects for the VACA were very small. We believe that it was possible through estimating the *α* for the VACA with heavy object three times larger than that for the VACA with light object (Table 7) so that the larger *α* for the heavy object could provide more active compliance and adjust the excess in the PLF.

**Table 8:** Mean (*n *= 20) PLF, peak acceleration and velocity with standard deviations in the parentheses for three different control strategies

Control strategy	PLF (N)	Peak acceleration (m/s^2^)	Peak velocity (m/s)
FACA (light object)	7.28 (0.27)	1.74 (0.12)	0.45 (0.07)
VACA (light object)	3.71 (0.11)	0.59 (0.06)	0.43 (0.05)
VACA (heavy object)	3.74 (0.13)	0.61 (0.08)	0.44 (0.6)

The results thus empirically prove that the VACA can be made effective to produce similar system characteristics for lightweight and heavy objects by customizing its parameters, which justifies Hypothesis II (see later for statistical significance). The results show that the VACA reduced the excess in the PLF and acceleration for both lightweight and heavy objects without significantly reducing the velocities, which indicate that the VACA can add compliance without sacrificing time efficiency.

### Improvement in HRI

**Fig. 12:**
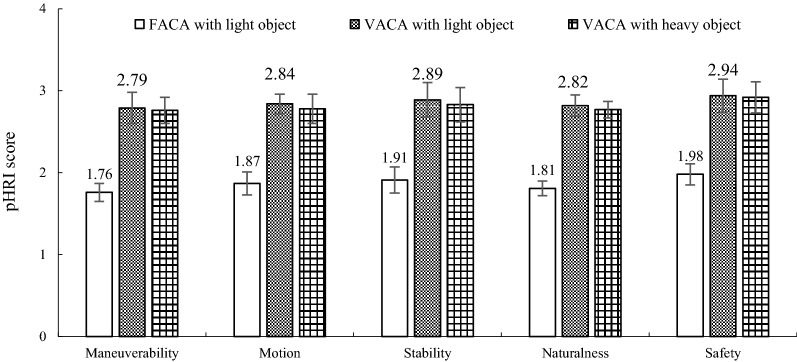
Mean (*n *= 20) pHRI evaluation scores for the three different control strategies

**Fig. 13:**
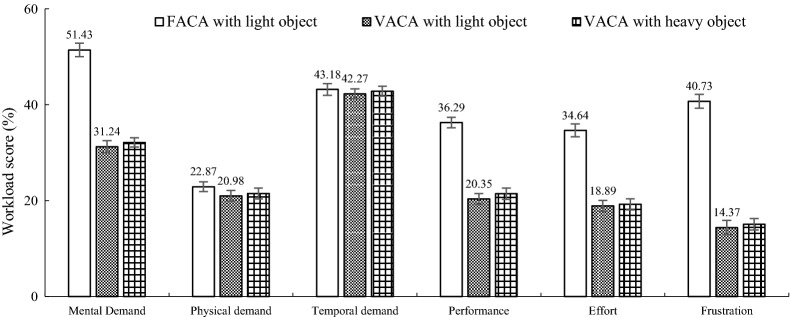
Mean (*n *= 20) cognitive workload rating (out of 100) for the six dimensions of the NASA TLX for the three different control strategies

**Fig. 14:**
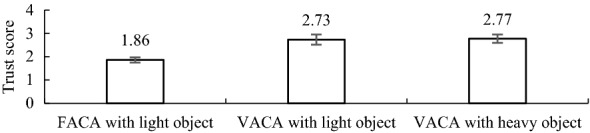
Mean (*n *= 20) human’s trust in the PARS for the three different control strategies

Based on Fig. 12, we calculated the average of evaluation scores of all pHRI parameters for the FACA and VACA with lightweight object separately. Then, we calculated the change (increase) in the average pHRI score between FACA and VACA and expressed the change in percentage. We followed similar calculation method to calculate the changes in cognitive workload and trust for the FACA and VACA. The results show that, on average, the pHRI improved by 53.05% (Fig. 12), cognitive workload reduced by 35.38% (Fig. 13), and human’s trust in the robot increased by 46.78% (Fig. 14) due to the VACA in comparison with the FACA for the lightweight object, which prove the impact of the novel VACA on HRI. The results in Figs. 12 and 14 show that the mean pHRI and trust scores are larger than 2, i.e., $M_{a} > 2$, $M_{o} > 2$, $S_{t} > 2$, $N_{a} > 2$, $H_{s} > 2$ and $T_{l} > 2$, for the VACA with lightweight object. We also found the mean $R_{j} < 0.2$ for the VACA with lightweight object. All these satisfy the soft constraints of the Algorithm 1. It means that the VACA enhanced the HRI as well as reduced the risk and thus helped achieve optimum HRI for the soft constraints. Figure 12 shows that the optimum perceived heaviness due to optimum mass values (*m*_1_ = 0.5 kg, *m*_2_ = 0.25 kg) produced high maneuverability and reduced the load force and acceleration, which resulted in satisfactory motion. High maneuverability and motion produced high stability, naturalness and safety. High maneuverability might also enhance the flexibility in the manipulation. It was proved in [19] that the VACA does not reduce perceived heaviness. Hence, it can be claimed that the VACA was effective to enhance the HRI as above without hampering user’s haptic perceptions at the time of manipulation.

Figure 13 shows that the mental demand and the demands for performance, effort and frustration reduced significantly probably due to the improvement in the motion through the application of the VACA. The low workload for physical demand was due to the advantage of power assistance. It did not vary too much for the three experimental conditions because the control parameter that resulted in haptic feelings (i.e., *m*_2_) did not change [19]. Again, the workload for the temporal demand did not change too much as the target manipulation time between the FACA and the VACA was similar. Low workload for performance and frustration for the VACA was due to the high pHRI achieved through the consideration of weight perception as well as customized variable admittance in the control system design. The workloads for mental demand and effort were low for the VACA, which also indicate the effectiveness of the VACA. The results thus can indicate that the improved pHRI due to the VACA (Fig. 12) could also contribute to improve the cHRI (Fig. 13), which show a tradeoff between the pHRI and the cHRI.

Figure 14 shows that the VACA improved the user’s trust in the assistive system for both lightweight and heavy object manipulation in comparison with that for the FACA. We posit that the active compliance through the VACA enhanced the motion, maneuverability, stability and safety in the manipulation that helped the users perceived better performance of the system. The system was also supposed to be less error-prone due to the advantages in motion, maneuverability, stability and safety provided by the VACA. We believe that all these increased the perceived trust of the users in the assist system for the VACA [20].

ANOVAs showed that variations in evaluation scores for pHRI (maneuverability, motion, safety, stability, naturalness) and cHRI (workload, trust) between the FACA and the VACA for the lightweight object were statistically significant (*p *< 0.05 at each case), which proved the positive impact of the novel VACA on the HRI. However, variations in pHRI (maneuverability, motion, safety, stability, naturalness) and cHRI (workload, trust) between the VACA for the lightweight and heavy objects were not statistically significant (*p *> 0.05 at each case). The results thus proved that the same control system and control parameters producing HRI for manipulation of lightweight object can also produce similar HRI for the manipulation of heavy object, though additional compliance needs to be provided to heavy object manipulation through adjusting some control parameter values (see Table 7). The results thus also justify Hypothesis II.

Again, the HRI for the lightweight object for the VACA was assumed as the optimum because we used optimum *m*_1_ and *m*_2_ values as the control parameters, and also provided additional compliance through variable admittance. These HRI were also intuitive and natural because the subjects used lightweight small-size object [18, 39]. We see as above that there is no statistically significant difference in HRI between lightweight and heavy object manipulation for the VACA. Hence, the HRI for the heavy object for the VACA can also be treated as intuitive and natural because these were calibrated with those for the lightweight object for the VACA. Thus, the results proved the effectiveness of the proposed natural and intuitive HRI calibration approach for heavy object manipulation with power-assist.

### Improvement in co-manipulation performance

#### Time efficiency

The mean approximate required times for a trial (lifting and releasing time for an object including the arrangement time between two consecutive lifts) for the three control protocols were determined separately using the recorded time data. Then, the time efficiency in the manipulation was calculated based on (*8*) for the three protocols separately. Table 9 compares the mean efficiency for the FACA with lightweight object to that for the VACA with lightweight and heavy objects. It is to be noted that the target time or speed depends on the required manipulation rate, operator’s own speed, fatigue and arrangement time including uncertainties between two consecutive lifts. High speed may adversely affect the HRI. Hence, an optimum tradeoff between time efficiency and HRI is desired.

**Table 9:** Mean (*n *= 20) co-manipulation performance for the three different control strategies

Control strategies	Value (%)	
	Efficiency	Precision
FACA with light object	94.29 (2.23)	95.84 (2.61)
VACA with light object	99.27 (1.76)	99.68 (1.67)
VACA with heavy object	99.23 (1.19)	99.73 (1.22)

#### Precision

The displacement profile in Fig. 8 for a trial for the FACA with lightweight object shows no deviation in the displacement, i.e., the measured position was almost equal to the targeted position (0.1 m), which indicates 100% precision of the manipulation based on (*7*). However, the displacement profile was multipeaked. It means that the subject initially lifted the object with high velocity probably due to high load force and acceleration, then sent the object slightly downward to reduce the velocity and the effects of high acceleration, and then completed the manipulation with a reduced velocity. This strategy helped maintain precision, but restricted higher efficiency due to adjustment of motion. For the trials when the humans did not produce multipeaked displacement, the efficiency might be higher, but the precision might be lower. The mean precision for the FACA with lightweight object for all trials was determined and compared to that for the VACA with lightweight and heavy objects as in Table 9.

Table 9 shows that due to the contribution of the VACA, the time efficiency on average increased by 4.98% and 4.94% for the lightweight and heavy objects, respectively, and the precision on average increased by 3.84% and 3.89% for the lightweight and heavy objects, respectively. This performance of VACA was achieved at the optimum HRI conditions (*m*_1_ = 0.5 kg, *m*_2_ = 0.25 kg). Hence, we posit that this performance is also the optimum. ANOVAs showed that both the time efficiency and the precision between the lightweight and heavy objects for the VACA did not differ much (*p *> 0.05 at each case), which proved that the controls produced similar performance for lightweight and heavy objects. The results thus justify Hypothesis II. The results thus also justify the natural and intuitive performance calibration approach. Again, in general, variations in each pHRI, cHRI and performance criterion between the subjects for the VACA for lightweight and heavy objects were not statistically significant (*p *> 0.05 at each case), which can indicate the generality of the results.

## Discussion

### Reliability and effectiveness of the results

Objective evaluation is always emphasized; however, there are some HRI criteria related to power-assist system that can neither be measured objectively nor be ignored. These criteria are necessary to evaluate the effectiveness of the power-assist system. This is why a portion of the results is based on subjective evaluations (e.g., Figs. 12, 13, 14) though we also used objective measurements (e.g., Tables 8, 9). However, subjective results are acceptable because (1) the subjective evaluations followed standard methods and metrics, e.g., NASA TLX, Likert scale [40–42]; (2) we used many subjects, determined the mean values of the subjective evaluation scores and also conducted the analyses of variances (ANOVAs) of the subjectively evaluated scores. The decision was not made based on one subject’s evaluation. Instead, it was made based on the mean or average values, and the ANOVAs were also taken into account to reach a decision (i.e., to understand the level of variation); (3) the subjective results were used in conjunction with the objective results as the mixed method for triangulation that helped cross-check the results and reach more reliable decisions; and (4) similar subjective results have been proven reliable in past cases [18–22].

We believe that the proposed evaluation system has been proven effective that we can understand through the obtained evaluation results and the ability of the results to satisfy the desired interaction characteristics, system behavior and performance. However, to make the evaluation system more effective, the following measures can be taken: (1) add more objective criteria as the evaluation criteria, (2) conduct a short survey with workers/engineers/researchers working with power-assist robots in industries to understand more useful and practical evaluation criteria, (3) conduct another survey based on studies to compare the evaluation scheme proposed in this article with the state-of-the-art evaluation approaches, (4) provide more trainings to the subjects about the evaluation criteria and methods to bring uniformity in the evaluation, etc.

### Flexibility and power assistance in object manipulation

As we described in “Introduction” section, flexibility in object manipulation means the manipulation method that can be easily modified to respond to altered circumstances or conditions. It may mean manipulation that is adaptable, adjustable, versatile, etc. [2, 3]. Flexibility is one of the desired outcomes of power-assisted manipulation [5, 19]. The presented manipulation was flexible that can be understood in the following ways:

1. The object weight was carried out by the robotic system, and the manipulation motion was provided by subject’s load force. Thus, the human could easily lift the object and hold or position it at any position within the planned path/trajectory. In addition, the subject could easily adjust the lifting velocity during manipulation as it was influenced by human subject’s input (load force). The presented lifting motion was 1 DOF (vertical up-down), and thus, the human could easily lift the object and hold or position it at any position along the vertical DOF within mechanical constraint. If the manipulator is upgraded to 6 DOF, the human can easily manipulate the object and hold or position it at any position/orientation within the specified 6 DOF space, which can further augment the flexibility.
2. In industrial applications, the same robotic system as proposed can be utilized to manipulate objects of different shapes, sizes, surface textures, weights, etc., by workers of different skill levels (e.g., skilled workers, semiskilled workers). This is possible because human with flexible dexterous skills is a part of the manipulation system and human can adapt with these situations [6], which can provide flexibility to the system. Note that here the manipulation method is itself flexible/adaptable. It is not necessary that the object is flexible/deformable, but deformable/flexible object can also be manipulated using a PARS [4].

In addition, a PARS can reduce haptically perceived weight to 40% (or less) of its actual weight as observed in [19]. This heaviness reduction in the manipulated object indicates power assistance [5].

### Statistical significance of the results and justification of the hypotheses

In “Weight perception-based dynamics model for manipulating objects with the PARS” section, we adopted Hypothesis I that says that perception of object weight due to inertia differs from the perception of weight due to gravity for manipulating objects with a PARS. We then derived the dynamics of human–robot collaborative manipulation based on this assumption. In fact, it was a strategy of rendering system dynamics that was assumed to be necessary to address the weight perceptual problem in power-assisted object manipulation [5, 19]. In this paper, we did not present any direct proof of this hypothesis. However, it can be proved through comparing control performance of power-assisted manipulation between weight perception-based and conventional control methods. In addition, experiment 1 shows that weight perception-based admittance control system helped achieve optimum/satisfactory HRI including low risk for a set of hard constraints. Experiment 2 shows that weight perception-based variable admittance control further augmented the HRI, performance and compliance and helped achieve optimum/satisfactory HRI including lower risk for a set of soft constraints. We posit that these empirical results are sufficient to prove the effectiveness of the adopted hypothesis.

Based on Hypothesis II, it is our expectation that the HRI and manipulation performance for power-assisted manipulation of both lightweight and heavy objects should be similar. If so, we can obtain naturalness and intuitiveness in heavy object manipulation with power-assist through calibrating naturalness and intuitiveness with that for lightweight object manipulation because lightweight manipulation is considered as natural and intuitive in psychology [5, 18]. The obtained results are in line with this expectation. Table 8 shows that the PLF, acceleration and velocity between lightweight and heavy object manipulation do not differ much. “Improvement in HRI” and “Improvement in Co-Manipulation Performance” sections show using ANOVAs that differences in HRI and performance between lightweight and heavy object manipulation for the VACA are not statistically significant, which is also in line with our expectation. We posit that such ANOVA results are sufficient to justify Hypothesis II.

### Safety, risk and the guidance of ISO/TS 15066

Experiment 1 shows that weight perception-based admittance control helped achieve optimum/satisfactory safety and low level risk. Experiment 2 shows that weight perception-based variable admittance control helped achieve better safety and lower risk [33–36]. Thus, this article directly contributes to safety enhancement and risk reduction for collaborative industrial robotic system, helps maintain a collaborative work environment and brings human and robot closer to collaborate for a common goal. We thus believe that the presented approaches are in line with the guidance of ISO/TS 15066, ISO 10218-1 and ISO 10218-2 [37]. It is possible that the power-assist device can move involuntarily when the operator does not apply any force due to incorrect gravity compensation, which can be a very serious safety matter that does not comply with ISO15066 [37]. This is why we searched and sorted out only one *m*_1_ and *m*_2_ pair out of 36 pairs that had correct gravity and inertia compensation so that we could avoid unsafe situations and comply with ISO15066 [37].

### System stability

The position/velocity-based admittance controllers may have instability issues when the rendered dynamics are significantly different from the actual dynamics of the system. However, the rendered dynamics for both lightweight and heavy objects were not significantly different from the actual dynamics of the systems. We used same *m*_1_ and *m*_2_ values, but we adjusted the values of some dynamic parameters for the systems for the VACA to adjust with lightweight and heavy objects (Table 7). Here, the potential instability was mitigated through utilizing the active compliance algorithm VACA, which was also adjusted for lightweight and heavy objects through adjusting some dynamic parameters (Table 7). This difference provided differential compliance to object manipulation with the same controller for the lightweight and heavy objects for the same *m*_1_ and *m*_2_ values. Furthermore, we investigated the instability issue two times during experiments: (1) in the first time using step voltage response in Fig. 11, (2) in the second time, the results in “Results of experiment 2” section were verified empirically by experiments that did not show stability problems for lightweight and heavy objects. Moreover, we previously determined the optimum control system parameter values such as *m*_1_ and *m*_2_ values that reflected appropriate inertia and gravity and made the system stable and compliant.

### Comparison with similar state-of-the-art approaches

The presented results are superior to the state-of-the-art results in terms of HRI and performance. The results are superior to that of the gravity compensation method in robot dynamics [6, 10, 14] because we did not use zero gravity that did not remove haptic feelings; instead, we optimized the feelings in power-assisted manipulation [22]. The results are also superior to that of the partly compensated gravity method [8, 12, 13] because we used optimized virtual mass value (*m*_2_) that provided optimized haptic feelings in the user [19, 22]. The results are also superior to the feed-forward model of the load force because such approach is uncertain that creates uncertainty in HRI and manipulation performance [7, 8]. Results of other approaches such as the model-based predictive controller [23] and constant torque/force method [24] are based on computed output forces and accelerations that may not fit with human user’s feelings and psychology, but the presented results can fit human feelings and psychology as the evaluation shows.

The results for the proposed weight perception-based FACA seem to be better than that of the state-of-the-art control strategies [6–17] because the proposed controllers are more human-friendly as evidenced through the presented HRI results. In particular, the VACA in [12, 13] did not consider the effects of excessive acceleration generating from user’s error in the programming of load force due to difference in perception between visual and haptic weights. Hence, the VACA in [12, 13] cannot guarantee optimum HRI and performance. In fact, an extensive evaluation scheme is absent in [12, 13]. The state-of-the-art PARSs [6–17] presented the evaluation partly, and the evaluation schemes were not comprehensive comprising of pHRI, cHRI and manipulation performance. The objectives of the state-of-the-art systems were to address one or few specific evaluation criteria. The proposed evaluation scheme herein is believed to be the only available evaluation scheme that is comprehensive. Thus, the proposed results based on the presented evaluation scheme are superior to the state-of-the-art results in terms of completeness, comprehensiveness and evaluation criteria and results such as the pHRI, cHRI and manipulation performance [6–17]. The proposed HRI optimization method is unique because no optimization method was proposed to optimize HRI and performance for the state-of-the-art systems [6–17]. Finally, calibrating naturalness and intuitiveness is totally novel, and such approach is not investigated in the state-of-the-art systems [6–17], except presented herein.

### Extrapolation to real scenarios in industries

The following two requirements need to be satisfied to extrapolate the obtained results to real scenarios in industries:

1. *Multi*-*DOF Dexterous Manipulation*: We here considered only 1 DOF manipulation, but 6 DOFs are required for dexterous manipulation in actual applications. We believe that we achieved our objective with 1 DOF simple manipulation, and similar approach/method can be applied to more complex multi-DOF systems for dexterous and robust manipulation. We emphasized 1 DOF manipulation along vertical direction because (1) the human feels the highest level of heaviness and thus may require the highest amount of power assistance for upward manipulation along vertical direction (against the gravitational weight of the object), and (2) such vertical lifting tasks are most common in industrial practices. In addition to vertical lifting, the weight perception-based control design approach can be considered for Cartesian manipulation along other two DOFs (left–right and forward–backward). The control design can also be extended to three rotational DOFs with respect to the three Cartesian DOFs. This can allow dexterous manipulation [51]. In addition, kinematically redundant DOFs can be utilized for other advantages in manipulation such as obstacle avoidance and safety [20]. In all these proposed cases, the system characteristics such as kinematics and kinetics may be analyzed, and the control parameters may need to be adjusted for each DOF depending on observed system characteristics for that DOF. Robot vision-based visual servoing and image processing [20], machine learning [52], passive compliance [49] and intelligent stochastic decision-making [53] can be incorporated in the proposed admittance control strategies to augment the overall performance and robustness of the system. The multi-DOF system can be evaluated with end users such as industry workers. In these ways, the system can be made more general that can also provide more versatile performance in real industrial applications.
2. *Robot Structure*: Multi-DOF robot structures suitable for dexterous manipulation targeting actual needs of concerned industries should be developed. The proposed PARS can be mounted on a mobile robotic base that can facilitate using the PARS in different locations of industry floors.

## Conclusions and future works

A novel method to reflect weight perception in the dynamics and control of a PARS for lifting lightweight objects was introduced in the form of a FACA, and a comprehensive scheme of evaluation and optimization of HRI and manipulation performance was proposed. Results showed that consideration of weight perception in dynamics and control was effective to achieve optimum HRI (and performance) for a set of hard constraints. Then, a novel VACA was proposed as an augmented version of the FACA to provide active compliance, and its effectiveness was first proved for manipulation of lightweight object and then validated for manipulation of heavy object with power-assist. Results showed that the VACA significantly improved the HRI and performance in comparison with that for the FACA for both lightweight and heavy objects and thus also helped achieve optimum HRI (and performance) for a set of soft constraints. The FACA and VACA results justify selecting *m*_2_ (gravity term) different from actual gravity term as well as from inertial mass term (*m*_1_). The results for the VACA did not significantly differ between lightweight and heavy object manipulation due to adjustment of the VACA parameters. The results thus justify the intuitive and natural HRI and performance calibration approach, which states that natural and intuitive HRI and performance for heavy object manipulation with power-assist can be achieved through calibrating this with that for manipulating lightweight object with power-assist for the same control system and the same control parameters, but with slight adjustment in the extent of active compliance. The proposed PARS design and controls are simple and the results are fundamental, but the results are novel and useful to develop controls of user-friendly power-assist devices for handling heavy objects in industries, e.g., manufacturing and assembly, construction, mining, logistics and transportation, timber, agriculture, and rescue and disaster operations. The results, in general, enrich the state-of-the-art knowledge in robotics, controls and human-centered flexible automation in industries.

In the near future, we will improve the optimization methods using advanced objective functions, weight factors and constraints. We will expand the system to multi-DOF system including rotational DOFs and the novel control design and evaluation will be expanded to 6-DOF dexterous manipulation, which will give the power-assist system a general platform and will help validate the effectiveness of the proposed methods with respect to power-assist robotic systems used in real industrial settings. We will increase the number of subjects and use different types of objects to increase the generality of the results. We will apply vision-based visual servoing to add additional intelligence to the VACA to distinguish objects of different sizes.

## List of symbols

$F_{x}$: friction force in the ball screw system for manipulation along vertical (*x*-axis) direction (N)
$f_{ax}$: actuation force along *x*-axis (N)
$f_{hx}$: load force (vertical lifting force applied by the human) along *x*-axis (N)
*G*: feedback position control gain
*g*: acceleration of gravity (m/s^2^)
$K_{x}$: viscosity of the linear slider along *x*-axis (N s m^−2^)
*m*: virtual mass for the manipulated object to be used in the control system (kg)
*x*: actual displacement of the lifted object along *x*-axis (m)
$x_{c}$: commanded displacement of the lifted object along *x*-axis (m)
$x_{d}$: desired displacement of the lifted object along *x*-axis (m)
